# Nonlinear self-calibrated phase-contrast correction in quantitative cardiac imaging

**DOI:** 10.1186/1532-429X-16-S1-P349

**Published:** 2014-01-16

**Authors:** Ek T Tan, Anja C Brau, Christopher J Hardy

**Affiliations:** 1 GE Global Research, Niskayuna, New York, USA; 2 GE Healthcare, Garching, Bavaria, Germany

## Background

Residual background phase in cardiac phase-contrast (PC) imaging introduces velocity errors that bias quantitative flow measurements [1]. While the bias can be offset using static phantoms [2], improved workflow is realized if self-calibrated correction is performed by fitting the phase of static tissue from the in vivo images. However, the residual phase can be nonlinear in space and the vessels of interest, e.g. great vessels, are often far from any static tissue in the image. This means that a linear fit [3] can sometimes result in under-fitting, while fitting with higher spatial-orders can result in over-fitting.

## Methods

We propose a nonlinear self-calibrated approach, which assumes a nonlinear shape. This follows observations that the residual phase is similar in shape to that of the concomitant field. Therefore as compared to linear fitting that uses 4 terms (constant + XYZ), the nonlinear-fit has 5 terms that also include the concomitant field. Further steps are taken to improve the fit, which include iterative removal of outliers that frequently occur at tissue boundaries, and weighting velocities from the quiescent cardiac phase more heavily to reduce effects from flow artifacts at systole. To prevent over-fitting, the corrected phase is weighted by an assigned weight, determined by the probability of the fitted phase exceeding previously proposed velocity specification limits of +/-6 mm/sec. In other words, if the fitted phase has only a small effect, no correction is done.

## Results

**Figure 1:**
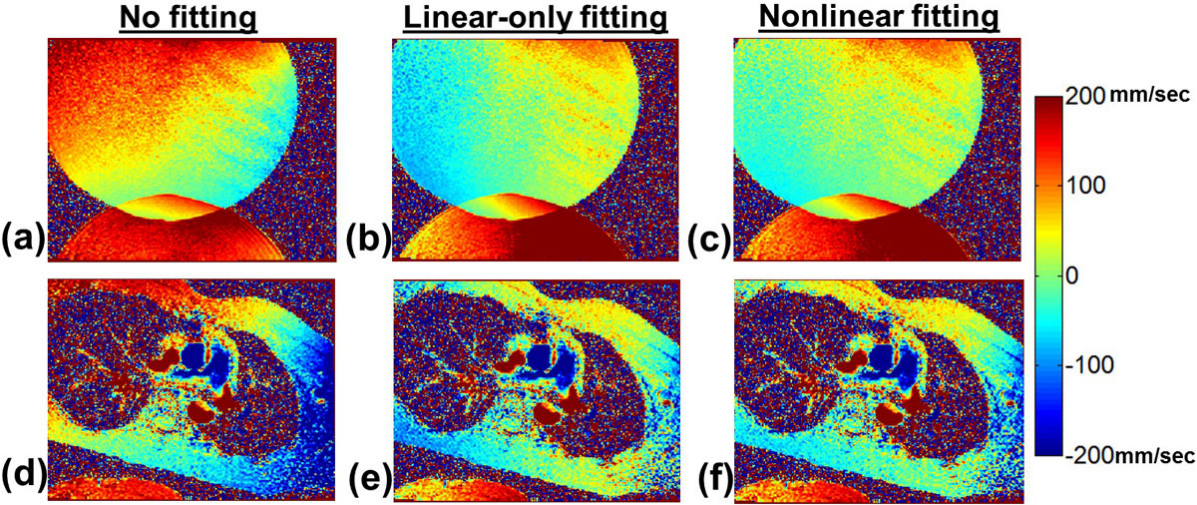
**Oblique 2D CINE-PC velocity maps from (a-c) static phantom and (d-f) in vivo scan, with no fitting applied (a,d), in-vivo-derived linear fitting only (b,e) and in-vivo-derived, nonlinear fitting (c,f)**. Linear fitting resulted in reduced residual velocity in static tissue and in the phantom, and further reduction in residual velocity was seen with the nonlinear fitting.

**Figure 2:**
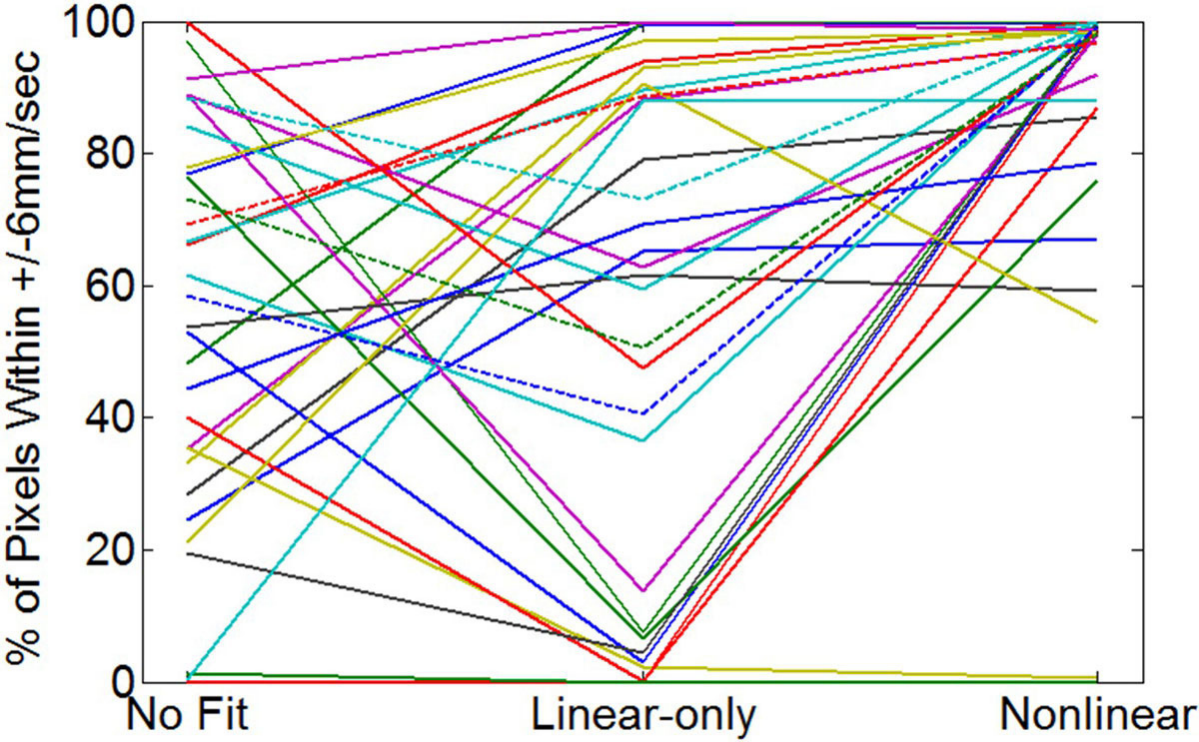
**Plots of percentage of pixels within +/- 6 mm/sec velocity limits as obtained with no fitting, linear-only and nonlinear fitting for 31 CINE phase-contrast images**. 27 images were acquired from 11 subjects at 3T (solid lines) and 4 images were acquired from 1 subject at 1.5T (dashed line). With no fitting, 4/31 instances had > 90% pixels within a 4-cm radius at isocenter that are within the velocity limits. With linear and nonlinear, 7/31 and 25/31 instances had > 90% of pixels within the velocity limits.

12 normal subjects were imaged at 3T and 1.5T with 2D CINE-PC using varying imaging parameters, yielding 31 PC images. Figure 1 shows results obtained using the proposed correction. A large static phantom was also imaged and phase corrected using the in-vivo-derived fits to provide a ground truth comparison. The difference in velocities between the fitted result and ground truth was measured within a 4-cm radius region at isocenter (near the great vessels). The percentage of pixels with velocity differences within +/-6 mm/sec are shown in Figure 2. Nonlinear was superior to both linear-only fitting and no fitting, with 25/31 instances having > 90% of pixels within the specified velocity limits.

## Conclusions

A self-calibrated, nonlinear phase-contrast correction method was demonstrated to provide superior results to linear-only correction. Residual motion in the static phantom and possible thermal drift may bias phantom results, and are also reasons for favoring self-calibrated correction. The theoretical basis for the nonlinear shape may lie in complex interactions with eddy-currents, which are not accounted for in the standard concomitant field correction. Further work involves validation on more scanners and with quantitative flow phantoms.

## Funding

NA.
